# Reply to Drs. Law and Williams

**Published:** 1901-07

**Authors:** J. P. Foster

**Affiliations:** Selby, S. D.


### CORRESPONDENCE.

### REPLY TO DRS. LAW AND WILLIAMS.

EDITOR JOURNAL OF COMPARATIVE MEDICINE, ETC.:

DEAR SIR: In the April issue of the JOURNAL, Drs. Law and Williams, both connected with the New York State Veterinary College, reply to my communication published in your March number. Dr. Law is perfectly right in the statement he makes, in which he claims that the first graduates of his school to pass the "whole of their period of professional study" in the New York State Veterinary College were the graduates of 1899. Dr. Law, however, misunderstands my meaning when I say "having passed the whole period of professional study in the three-year school." I used the expression "the three-year school" in the same sense in which one might refer to "the stage" or "the race-track," and did not mean that the sudent had necessarily put in all his professional study at one particular three-year school. I knew the condition of affairs at Cornell with regard to the matters Dr. Law mentions, and certainly would have been very foolish to have made statements that would not bear investigation, as I rather expected that my communication would be answered. I could probably have made by meaning clearer had I said "in *a* three-year school." What I meant was this, for example: A student attends the New York College of Veterinary Surgeons at New York City during the seasons of 1895–96 and 1896–97 (which was a three-year school at that time) and during the session of 1897–98 takes a third year at Cornell. In the same class there is an Ontario graduate who captures the Horace K. White prize at graduation. Did not the Ontario graduate successfully compete with the student who has "passed the whole period of his professional study in the (or *a*) three-year school?" I think Dr. Law will admit that the example mentioned is tenable, and I will ask him if this very circumstance has not happened? As Dr. Law has seen fit to mention the names of certain students in his reply to my communication, he probably will not object to my doing the same. Therefore, in order to justify my position and dispute some of the assertions of Dr. Law, I shall attempt to make a rough analysis of the alumni of the New York State Veterinary College.

The first class to graduate was the one of 1897. It was composed of Drs. Ryder and Howe (Ontario graduates of 1896) and Dr. Weihe. I do not know what veterinary school Dr. Weihe attended previous to this time. He certainly must have put in two years at some veterinary school in order to be entitled to the advanced standing that he secured, and it seems to me that this must have been a three-year school, because he was not a graduate at the time he entered Cornell in the fall of 1896. Therefore, he had either put in two sessions in a three-year school or had completed his course in a two-year school, but did not receive his degree. This latter proposition is hardly probable. Possibly Dr. Law can explain this. Drs. Ryder and Howe were the "prize men" of the class. They did not have much competition, to be sure; but the "other fellow" had a chance to beat them if he could. At Ontario prizes are given in each subject examined upon. They usually consist of first, second, and third prize. Then come "honors" for those passing above a certain mark. Out of five subjects (constituting the entire examination at that time) Dr. Ryder received a prize on one subject and failed to get even "honors" in two subjects at his junior examination. Dr. Howe got no prize, and failed to get "honors" in two subjects at his junior examination. Drs. Ryder and Howe made the following record at their senior examinations: Out of seven (including dissected specimen) subjects, which constituted all the branches examined upon, Dr. Ryder received two prizes and failed to get even "honors" in materia medica. Dr. Howe received no prize and failed to get "honors" in two subjects. In stating these facts I refer to the published prize list which appeared in the annual announcement of the Ontario Veterinary College for the years mentioned.

The next class to graduate at Cornell was that of 1898. In this class were four students, one of whom was Dr. Stanclift, an Ontario graduate of 1896, who won the Horace K. White prize. His Ontario record is as follows: As a junior he won three prizes, but failed to get "honors" in physiology. As a senior he won three prizes, and received "honors" in everything. In the class of 1898 was Dr. Dustan, who had previously attended the New York College of Veterinary Surgeons (I am in possession of the announcement of this school in which Dr. Dustan's name appears in the list of matriculates of 1896–7), and it is fair to presume that he as well as Drs. Kelly and Lehrman had passed as many as two sessions of professional study elsewhere before starting in at Cornell, else why did they get the advanced standing that they were allowed? It is possible that Drs. Kelly and Lehrman spent two sessions at Cornell and one session previous to their entering Cornell in some other college. In any event, it is highly probable that Drs. Dustan, Kelly, and Lehrman passed the whole of the period of their professional study in "three-year schools."

Drs. Ryder, Howe, and Stanclift were all good students at Ontario; certainly far better than the average student; but Dr. Law is mistaken in the idea that they all took prizes at Ontario. Neither of the gentlemen mentioned won the "gold medal" at Ontario, and Dr. Ryder was the only one of the three that was among the seven students chosen to compete for the "gold medal" (according to the Ontario prize list).

This brings us up to the "crack" class of 1899. Dr. Law says: "In 1899 the students who entered the New York State Veterinary College when it first opened its doors in 1896 were eligible for graduation, and for the first time these came into competition with graduates of the Ontario Veterinary College who had spent one year at Cornell, and neither then nor since has any graduate of the Ontario Veterinary College been fortunate enough to carry off a Horace K. White prize." Now, let us see of whom this 1899 class was composed. It consisted of six students, two of whom were already graduates. Dr. Fish graduated from the Veterinary Department of Columbian University in 1896, and Dr. Scammel graduated from Ontario in 1896 and from the Veterinary Department of Columbian University in 1897. It will be noticed that up to this time all the Ontario graduates taking a third year at Cornell were from the Ontario class of 1896. Dr. Scammel did not take a prize or appear in the "honor list" in a single study during the two sessions he was at Ontario. According to the annual announcement of the New York College of Veterinary Surgeons Dr. Perkins (of this Cornell class of 1899) was a matriculate during their session of 1896–97. We now have three students to account for, Drs. Gay, Illston, and Potter, and the student or students who entered Cornell as freshmen "when it first opened its doors in 1896" must of necessity be among these three.

The class of 1900 was composed of seven students, among them being one Ontario graduate, Dr. Corrigan (Ontario, 1899), a personal friend of mine. I should like to ask Dr. Williams if Dr. Corrigan did not prove a very apt student in the branch of surgery? How many of those students who entered Cornell "when it first opened its doors in 1896" received higher marks in surgery?

Dr. Law points with great pride to the fact that beginning with the class of 1899 no Ontario graduate has been able to successfully compete with a "home product" whose education represents twenty-seven months of collegiate work. There is little to "crow" about in this fact. It would certainly be strange that if two students of the *same* mental calibre, devoting an *equal* amount of application to their studies, were to compete for honors that the student representing twenty-*one* months (twelve at Ontario and nine at Cornell) professional study could excel the student who had spent twenty-*seven* months in professional study.

I do not care to argue that "black is white," or that, as I remarked in my former communication, *everything else being equal*, twelve months of study is as good as twenty-seven. I did say that the course at Ontario was as good, and, I think, probably far better, than the courses in some of the three-year schools. I made reference to the fact that in some of the veterinary colleges that might be mentioned many things that are advertised in the line of professors of certain subjects, "laboratory exercises," etc., fail to materialize. I hope that Dr. Williams will "back me up" in my statement in regard to these matters, and I refer him to his article in the *American Veterinary Review*, April, 1900, p. 15.

Dr. Law makes the following statements in regard to my communication: "He follows the too common course of stating an apparent truth in such a way as to convey an entirely false impression to the reader." Dr. Law also says: "Which should be corrected in the interest alike of truth and veterinary education." Now, this is pretty "strong talk," and if a person was very sensitive and allowed small matters to bother him he might become offended; but I feel charitably inclined toward the "good, kind doctor," because I realize that at the time he wrote his reply to my article he was suffering from a complication of grievances. The editor of the JOURNAL is an accomplice of mine in being the author of Dr. Law's perturbation of spirit. In publishing the obituary of Prof. W. Williams, of Edinburgh, the JOURNAL failed to state that "James Law got a silver medal, too." Probably very few readers of the JOURNAL are interested in knowing just what metal those medals were composed of. The good taste displayed in taking advantage of a discussion of this kind for the purpose of publishing the fact that he (Dr. Law) "tied" Prof. Williams (deceased) for "best general examination" in 1857 may be questioned by some.

Dr. Law attempts to misconstrue my sentiments regarding educational standards, and as he has accused me of attempting to distort the truth I can only make use of the somewhat threadbare expression "there are others."

The "gold medalist" of 1898 at the Ontario Veterinary College holds the position of "assistant in physiology and materia medica" at the New York State Veterinary College. He is also a student at the college. Is it not strange that Dr. Law cares to have such a "poorly educated" teacher on the staff of his school?

Dr. Williams refers to my "*writing up*" the Ontario Veterinary College, which is certainly the "unkindest cut of all." After patiently reading slur upon slur, directed against my alma mater, and because I make a feeble attempt to dispute the inference that all Ontario graduates are "poorly educated" and "illy equipped for the practice of their profession," because they graduated from "schools which could grant diplomas on short notice, easy terms, etc.," I am charged with "writing up" the Ontario College.

Dr. Williams' argument in regard to the teaching of surgery is *all right.* I have no disposition to evade the issue, and will admit that what Dr. Williams contends is proper. I merely referred to his remarks in my first communication as an example of the attacks upon the Ontario College, emanating from certain quarters, and stated further that some of the Ontario graduates had become very capable and well-known, although their knowledge of surgery was gained in a manner condemned by Dr. Williams. Dr. Williams is a graduate of what has always been a three-year school, and probably has a better right "to talk" than some others that might be mentioned.

At the risk of getting into more trouble, I shall refer to the article read by Dr. Austin Peters at the meeting of the American Veterinary Medical Association in Detroit last September. This article was entitled "Obstacles to Enforcing Regulations Requiring the Tuberculin test in Inter-State Cattle Traffic," and was published in the November issue of the JOURNAL. At the bottom of page 676 the following appeared: "Numerous specific instances of dishonest work might be given. Last summer an Ontario graduate, supposed to be one of the leading veterinarians," etc. Then follows an account of his dishonest work in applying the tuberculin test. I should like to inquire where the connection is between Ontario graduates, honesty, and the tuberculin test? Was it necessary, in order to have his audience understand this article thoroughly, that Dr. Peters should designate the school from which this man graduated? I was not aware that any one school had a monopoly on the virtue of honesty in its alumni.

In closing, I shall refer to the last paragraph in Dr. Law's communication, in which he says, in referring to teachers of Ontario, "that they have not accomplished more is due to the system," etc. I suppose all teachers feel that the success of their students is the proof that they (the teachers) have accomplished something, and I do not think that the teachers at the Ontario Veterinary College have cause to feel ashamed of their efforts. At the risk of having Dr. Williams accuse me of "writing up" the Ontario Veterinary College, I will present a few facts that I have recently investigated and which I believe to be correct.

Twenty-six States in the United States maintain the office of State Veterinarian. Out of this number 12, or over 46 per cent., are graduates of Ontario.

Of the 378 present members of the American Veterinary Medical Association 91, or over 24 per cent., are graduates of Ontario (including the President and one of the Vice-Presidents).

Of the 44 State secretaries of the American Veterinary Medical Association 11, or 25 per cent., are graduates of Ontario.

On page 568, September (1900) number of the JOURNAL, in list of recent promotions in the Bureau of Animal Industry, over 25 per cent. of those mentioned are graduates of Ontario.

On page 774, December (1900) number of the JOURNAL, in list of recent promotions in the Bureau of Animal Industry, about 28 per cent. of those mentioned are graduates of Ontario (an Ontario graduate receiving the highest salary).

On page 76, April (1901) number of the *American Veterinary Review* it will be noticed that out of the 27 inspectors employed in the Federal Meat-inspection Service at Kansas City, 7, or nearly 26 per cent., are Ontario graduates.

I have no statistics relating to the Federal Meat-inspection Service in the cities of Buffalo, Milwaukee, and St. Joseph, but know that there are a number of Ontario graduates employed in the service at these points.

I believe that the Ontario graduates are "holding down" their share of the positions at the different experiment stations and agricultural colleges in the United States and Canada. As I have already given a list of the veterinary colleges in which Ontario graduates are members of the teaching staff in my former communication, I will not mention them in this reply.

Very respectfully,

J. P. FOSTER.

SELBY, S. D., May 4, 1901.

